# Improvement of Mechanical Properties of Composites with Surface Modified B_4_C for Precision Machining

**DOI:** 10.3390/ma16020882

**Published:** 2023-01-16

**Authors:** Jun Ding, Jintao Wang, Hao Yang, Zhenglong Liu, Chao Yu, Xiangcheng Li, Chengji Deng, Hongxi Zhu

**Affiliations:** The State Key Laboratory of Refractories and Metallurgy, Wuhan University of Science and Technology, Wuhan 430081, China

**Keywords:** molten salt method, Nano (Zr, Ti)B_2_, coating, B_4_C–(Zr, Ti)B_2_ composite ceramics

## Abstract

In order to solve the problem of difficult sintering and high brittleness of B_4_C-based ceramics, B_4_C@ZrB_2_-TiB_2_ composite powder was synthesized by molten salt method, and B_4_C–(Zr, Ti)B_2_ composite ceramics were successfully prepared by spark plasma sintering. The effects of different raw material ratios on the composition, microstructure, and mechanical properties of the prepared composite ceramics were characterized by XRD, XPS, SEM, and TEM. The results show that ZrB_2_ and TiB_2_ were grown on the surface of B_4_C by template mechanism to form a dense nanocrystalline coating, and the original surface of B_4_C was exposed gradually with the decrease of the ratio of metal powder. When the composite powders were sintered at 1700 °C, ZrB_2_ and TiB_2_ formed a solid solution, which can refine grains and improve strength. When the raw material ratio is n(B_4_C): n(Zr): n(Ti) = 12:1:1, the composite ceramics have excellent comprehensive properties, the Vickers hardness reaches 41.2 GPa.

## 1. Introduction

Given the higher grinding requirements of rough wafers grinding in the integrated circuit field, it is urgent to develop ultra-hard ceramics with excellent mechanical properties to meet the new growth requirements. The hardness of boron carbide ceramics is third only to diamond and cubic boron nitride, and the lower cost makes it have great application potential. Boron carbide has the characteristics of high hardness, high melting point, high modulus of elasticity, low thermal expansion coefficient and good chemical stability [1,2], and is widely used in mechanical equipment, abrasive abrasives, microwave absorption, and refractory antioxidants and other fields [3,4,5]. Zamora et al. [6] prepared dense boron carbide composites at low temperatures and they showed good wear resistance with only slight wear after linear sliding over long distances. However, the lower sintering temperature and the introduction of more low-hardness phases make it difficult to meet the grinding needs of rough wafers. The mechanisms of porosity elimination, grain boundaries, and volume diffusion of ceramics only play a role when the sintering temperature reaches more than 2000 °C This ultimately makes it difficult to achieve the sintering densification of B_4_C ceramics with poor plasticity (K_IC_ is about 2 MPa∙m^1/2^), and its ultra-hard properties are difficult to fully achieve [7,8].

In order to achieve the densification and sintering of B_4_C-based ceramics, various sintering aids and second phases were introduced to reduce the sintering temperature. It has been found that carbides and borides such as SiC, TiC, HfC, TaB_2_, TiB_2_, and ZrB_2_ benefit the performance improvement of composite ceramics [9,10,11]. Chen et al. [12] found that ZrB_2_–ZrC–B_4_C composites have been successfully fabricated by SPRS from B_4_C and different content of Zr, while the flexure strength and hardness decrease first and then increase with increasing Zr content. It is found that boron carbide containing 66% Zr has the highest fracture toughness (5.83 MPa·m^1/2^) and flexure strength (386.45 MPa) due to the uniform distribution of ZrB_2_ and ZrC. Sha et al. [13] explored the effect of carbon content on the mechanical properties of reaction-sintered B_4_C composite ceramics. When the carbon content is 10%, the composite ceramics have the highest bending strength (444 MPa) and elastic modulus (329 GPa). After adding an appropriate proportion of carbon and silicon in the preparation of B_4_C/TiB_2_ composite ceramics, Zhu et al. [14] found that layered graphite appeared at the grain boundary of B_4_C and it could react with B_2_O_3_ to cause volume shrinkage, which significantly improved the mechanical properties of the material. Yan et al. [15] introduced TiSiC_2_ additive to prepare B_4_C-TiB_2_ composite ceramics. Compared with the pure B_4_C ceramics prepared by the same method, the hardness of the composite ceramics decreased, but the flexural strength and fracture toughness increased significantly. Ren et al. [16,17] used NaCl–KCl as a molten salt medium to prepare the composite powder with Al_3_BC and TiB_2_ coating uniformly on the surface of B_4_C particles. Due to the conductivity of boride coating, the material transportation is dynamically enhanced through SPS sintering.

Spark plasma sintering (SPS) is a new rapid densification technology that uses high-frequency and high currents through the sample to heat the sample and apply mechanical pressure [18,19,20,21]. Yavas et al. [22] found that SPS sintering has higher surface energy than B_4_C powder with small particle size (HS) at heating rate of 75, 150 and 225 °C·min^−1^, which improves the sintering driving force and enables the sintering of B_4_C ceramics to be completed at 1590 °C. Moshtaghioun et al. [23] combined high-energy ball milling, annealing treatment and SPS to provide sinterability for ultrafine B_4_C powder, and the optimal sintering conditions were 100 °C∙min^−1^ temperature to 1700 °C for 3 min, which could prepare B_4_C ceramics with ultra-fine grains with a relative density of more than 98.5%. At the same time, due to the significant decrease of grain size and the increase of transcrystalline fracture mode, the ceramic has an ultra-high hardness of 38 GPa without corresponding ductile loss (~3 MPa∙m^1/2^). Experiments show that the two-step SPS treatment enables B_4_C powder to be densified while retaining nanoscale particles, with ultra-high hardness and good toughness [24].

Although many studies have improved the performance of B_4_C ceramics to varying degrees, there are still problems such as high sintering temperature, poor fracture toughness and lower hardness. In this paper, B_4_C–(Zr, Ti)B_2_ composite ceramic with high hardness was prepared using the SPS and using (Zr, Ti)B_2_–coated B_4_C powder prepared by molten salt as raw materials. The effects of the ratio of raw materials on the phase composition and microstructure of composite powders and ceramics were discussed, and the hardness of the ceramics was compared.

## 2. Materials and Methods

### 2.1. Materials

The composite powder for preparing composite ceramics was synthesized by molten salt method using B_4_C (Mudanjiang Diamond Co., Ltd., W1.5, Mudanjiang, China), ZrH_2_ (ST-NANO, 0.5 μm, Shanghai, China), TiH_2_ (ST-NANO, 0.5 μm, Shanghai, China) as raw materials, and NaCl (Sinopharm Chemical ReagentCo., Ltd, AR, Shanghai, China), KCl (Macklin, AR, Shanghai, China) as a molten salt medium. First, mixed the raw materials in the mortar for about 25 min (according to the mass ratio of m (mixed salt):m (mixed powders) = 7:3 and m (NaCl):m (KCl) = 3:2. ZrH_2_ and TiH_2_ will decompose and precipitate H_2_ at low temperatures to obtain metal Zr and Ti [25]. The actual reaction occurs between Zr/Ti and B_4_C. Due to the extremely low H content and the low addition of raw materials in each group of mixed powders, the ratio was calculated according to the molar mass of Zr and Ti. The molar ratio of B_4_C to Zr/Ti are 8:1:1, 12:1:1 and 16:1:1, named ZT8, ZT12, and ZT16, respectively. Put the mixture into the alumina crucible with a lid, then the crucible containing the mixture was placed in an atmosphere furnace and held to 1100 °C for 2 h under Ar atmosphere. The solid obtained after heat treatment was washed with deionized water after ultrasonic cleaning of 15 min in a water bath at 45 °C (these operations should be repeated three times). Lastly, put the products at 110 °C and dried for 12 h to obtain composite powder.

Accurately weighed composite powder was placed in a special cylindrical graphite crucible (Φ20 mm), and the crucible containing the powder was placed in an SPS sintering furnace for sintering (the inner wall of the crucible, and the composite powder were isolated with clean graphite paper, and the powder was compacted and sealed with a graphite plunger at the upper and lower ports). The sintering system of this experiment was: holding at 1400 °C for 3 min, holding at 1700 °C for 6 min, the heating rate was 100 °C/min, and the sintering pressure was 50 MPa. Pressure was increased gradually during heating and reached maximum pressure before maximum temperature. After the heat preservation, the pressure was gradually removed and the samples demoulded.

After removing the graphite paper from the ceramic samples, the ceramic samples were ground, polished, then characterized and tested for the performance of the samples.

### 2.2. Characterization and Testing

**Figure 1:**
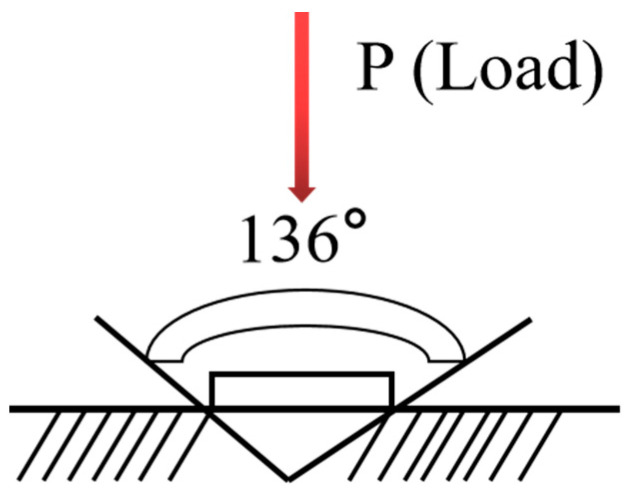
Indentation diagram.

The phase composition and microstructure of the composite powders and the composite ceramics were characterized using the X-ray diffractometer (XRD, X’Pert Pro, Philips, The Netherlands), field emission scanning electron microscope (SEM, Nova nano 400, FEI, Hillsboro, OR, USA) equipped with an energy dispersive spectrometer (EDS, IE350 Penta FET X-3, Oxford, UK) and high resolution transmission electron microscope (TEM, JEM2100, JEOL, Tokyo, Japan). The element composition and chemical bond bonding state on the surface of the composite powder was analyzed by an X-ray photoelectron spectrometer (XPS, AXIS SUPRA+, Shimadzu-Kratos, Hadano, Kanagawa, Japan). The Vickers hardness of the ceramic samples was measured by an automatic micro/macro hardness tester, the applied load was 0.5 kg and the loading time was 15 s. The indentation diagram is shown in Figure 1.

## 3. Results and Discussion

### 3.1. B_4_C@ZrB_2_–TiB_2_ Composite Powder

**Figure 2:**
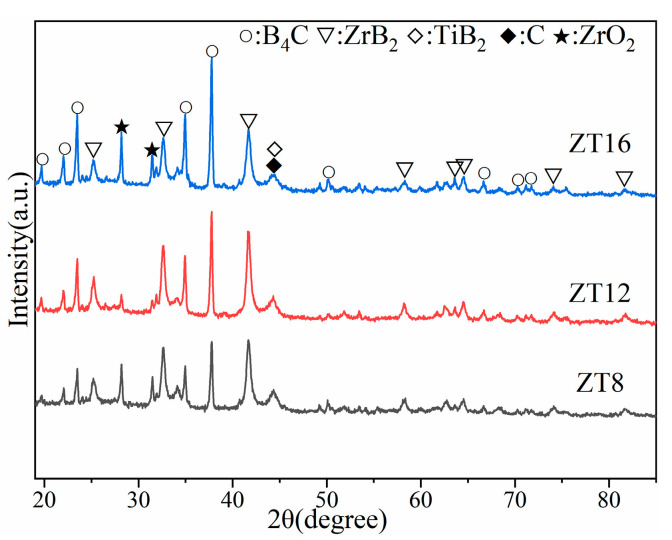
XRD patterns of the composite powder prepared at 1100 °C.

Figure 2 shows the XRD patterns of the composite powder prepared at 1100 °C. It can be seen from Figure 2 that the main phase of the composite powder is composed of B_4_C, ZrB_2_, and a small amount of ZrO_2_. The oxidation of metal zirconium powder and the reaction of B_2_O_3_ on the surface of B_4_C with Zr powder may cause the diffraction peak of ZrO_2_. Meanwhile, only a weak diffraction peak of TiB_2_ was detected in the composite powder, which might be the dissolution of TiB_2_ in ZrB_2_ [26], and the significant atomic number of Zr leads to the reduction of X–ray scattering factor of TiB_2_ [27].

**Figure 3:**
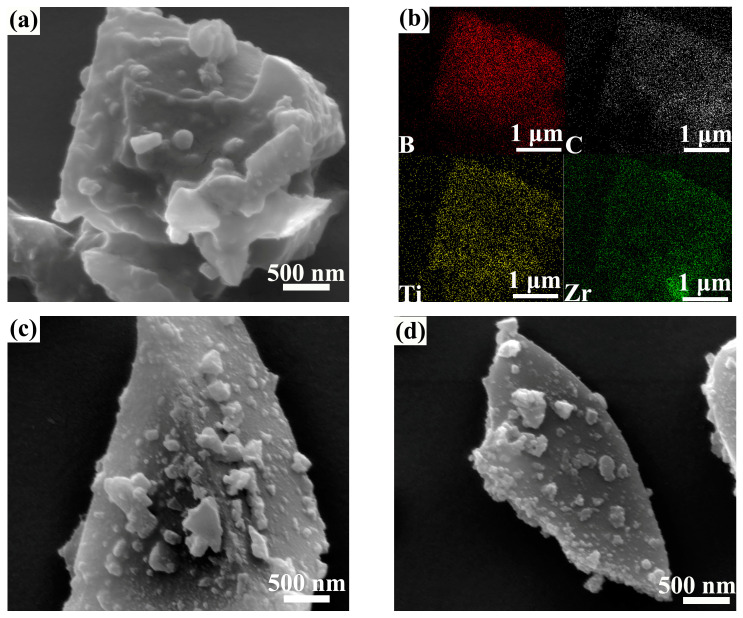
SEM images of the powder prepared at 1100 °C: (**a**) ZT8; (**b**) elemental mapping analysis in (**a**); (**c**) ZT12; (**d**) ZT16.

Figure 3 shows the SEM images of the composite powder prepared at 1100 °C. Figure 3a,b show the morphology of the ZT8 composite powder and its elemental mapping analysis. On the one hand, it confirms the generation of TiB_2_ and ZrB_2_ on the surface of B_4_C particles after heat treatment at 1100 °C in molten salt. On the other hand, it illustrates the homogeneous encapsulation of the two phases on the B_4_C particles. Figure 3c,d show the ZT12 and ZT16 composite powder morphology. The composite powder particles maintain the original shape of the irregular polyhedra of the B_4_C particles, and the original surface is gradually exposed with a decrease in the proportion of metal powders.

**Figure 4:**
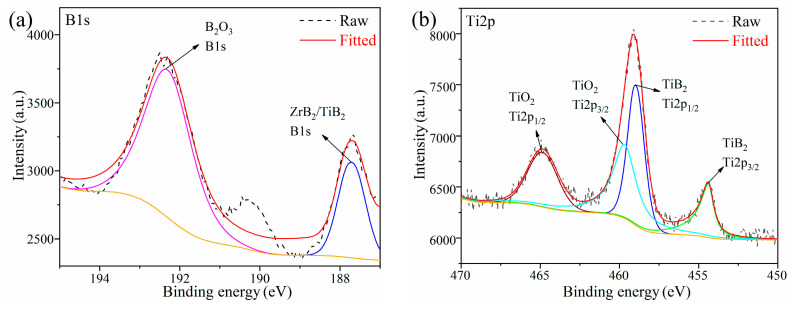
XPS patterns of the ZT8 powder prepared at 1100 °C: (**a**) B1s; (**b**) Ti2p.

To further verify the formation of ZrB_2_ and TiB_2_ on the surface of B_4_C particles, the chemical composition of ZT8 composite powder was analyzed by XPS. Figure 4a shows the pattern of B1s, which is divided into two peaks at 187.45 eV and 192.55 eV. Since the energies of the B–Zr bond [28,29] of ZrB_2_ and the B–Ti bond [30] of TiB_2_ are similar, the superposition of peaks may occur at 187.45 eV. The 192.55 eV corresponds to the B–O bond of B_2_O_3_ [31], which is the oxidation on the surface of the composite powder. The pattern of Ti2p is divided into four peaks, which are two peaks generated by the energy level splitting of the Ti–B bond and Ti–O bond in Figure 4b. The Ti2p_3/2_ sub–peak at 454.38 eV and the Ti2p_1/2_ sub–peak at 458.95 eV correspond to the Ti–B bond of TiB_2_ [32], which proves the existence of TiB_2_ and the coating of the surface of B_4_C particle. The Ti2p_3/2_ sub–peak at 459.67 eV and Ti2p_1/2_ sub–peak at 464.86 eV correspond to the Ti–O bond in TiO_2_ [33], due to the oxidation on the surface of the composite powder.

### 3.2. B_4_C–(Zr, Ti)B_2_ Composite Ceramics

**Figure 5:**
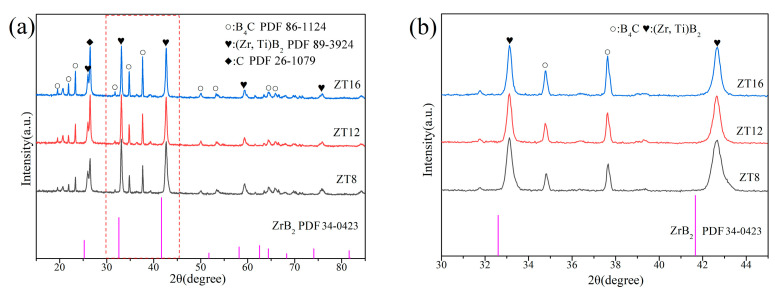
XRD patterns of the composite ceramics: (**a**) different samples; (**b**) enlarge image.

Figure 5 shows the XRD patterns of the composite ceramics sintering at 1700 °C. It can be seen from the picture that the primary phases are B_4_C (PDF #86-1024), (Zr, Ti)B_2_(PDF #89-3924), and C(PDF #26-1079). The diffraction peaks of ZrB_2_ (PDF #34-0423) shifts to high-angle azimuth (Figure 5b). As a result of the small size of Ti atoms, the solid solution replaces the Zr atoms into the lattice of ZrB_2_, causing distortion and compressive stress, resulting in the reduction of cell parameters (0.3100 nm) [27]. The diffraction peaks of ZrO_2_ disappear after high–temperature sintering. The lowest temperatures required for the complete conversion of Zr and Ti oxides into diborides were 1620 °C and 1690 °C under 0.8 CO ambient pressure. Furthermore, excessive B_4_C and lower CO partial pressure will further reduce the reaction temperature [34]. Asl et al. [35] found that ZrO_2_ will react with B_4_C to form a delicate ZrB_2_ connecting phase until it completely reacts in the subsequent heating process at 1281 °C.

**Figure 6:**
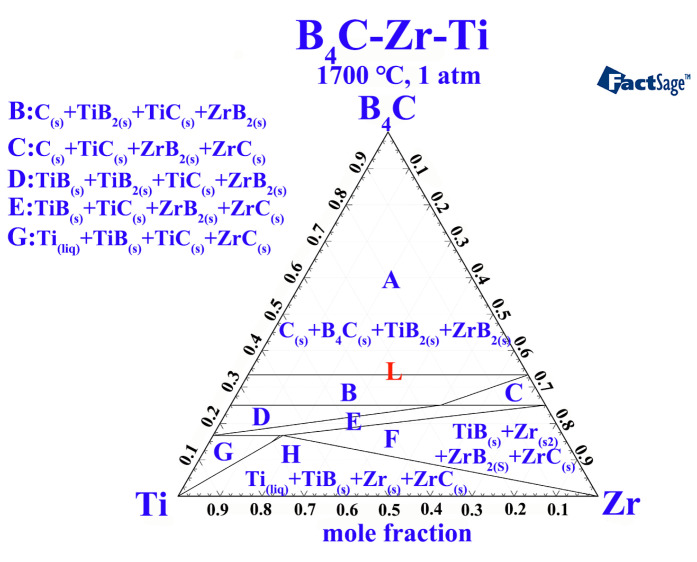
The ternary phase diagram of the B_4_C–Zr–Ti system at 1700 °C.

Figure 6 shows the ternary phase diagram of the B_4_C–Zr–Ti system at 1700 °C obtained by FactSage calculations. When the B_4_C content is high, the region above the phase line L is a stable four–phase composition of B_4_C, ZrB_2_, TiB_2_, and C at 1700 °C. Combined with XRD analysis, ZrB_2_ and TiB_2_ will generate (Zr, Ti)B_2_ solid solution after sintering at 1700 °C.

**Figure 7:**
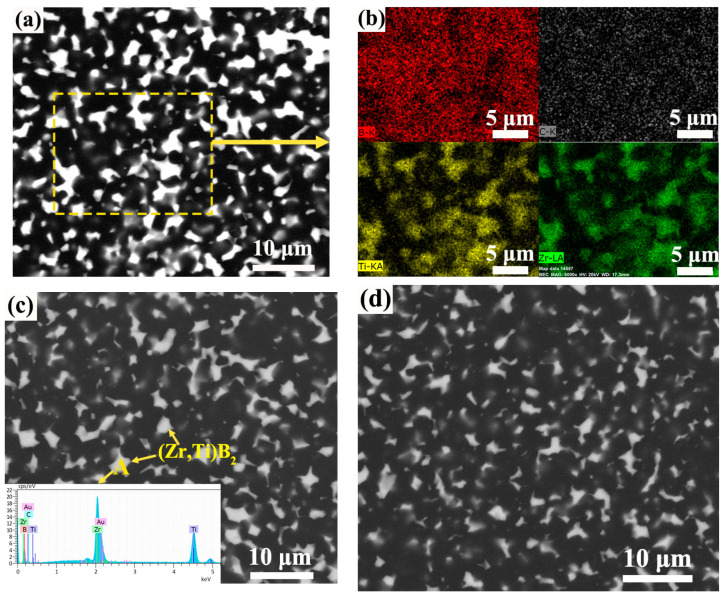
BSE images of the B_4_C–(Zr, Ti)B_2_ composite ceramics: (**a**) ZT8; (**b**) elemental mapping analysis of rectangular area in (**a**); (**c**) ZT12; (**d**) ZT16.

Figure 7 shows the BSE images of the B_4_C–(Zr, Ti)B_2_ composite ceramics with different raw material ratios. Due to the larger atomic number of Zr and Ti atoms show a brighter white lining in the backscattering mode compared to B and C atoms. Combining the XRD analysis in Figure 5 and the EDS analysis (in Figure 7b,c), it can be found that the bright white phases in Figure 7 are (Zr, Ti)B_2_. Figure 7a,b show the BSE image of the ZT8 sample and mapping analysis of the rectangular area. Zr and Ti elements are concentrated and evenly distributed in the bright white area. Furthermore, the same distribution interval of Zr and Ti elements and the shift of the XRD diffraction peak (Figure 5) confirms the generation of the solid solution of ZrB_2_ and TiB_2_. Figure 7c,d are the BSE plots of ZT12 and ZT16 samples, respectively. With the decrease of the additional amount of Zr and Ti, the distribution of (Zr, Ti)B_2_ in the B_4_C matrix was gradually sparse and dispersed.

**Figure 8:**
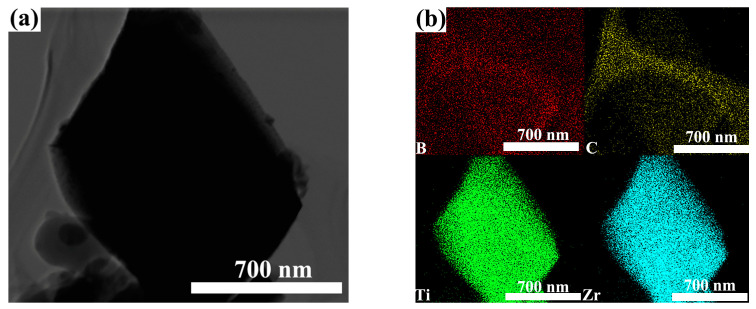
TEM images of composite ceramic: (**a**) ZT8; (**b**) elemental mapping analysis of (**a**).

Figure 8 shows the TEM image of ZT8 composite ceramic at 1700 °C. Figure 8b shows the elemental mapping analysis of Figure 8a. The surface of the ceramic sample shows the aggregation of B, Zr and Ti. Further illustration of (Zr, Ti)B_2_ solid solution formation and good bonding with B_4_C grains. The segregation of the C elements in the grain is mainly due to the influence of carbon supporting film.

**Figure 9:**
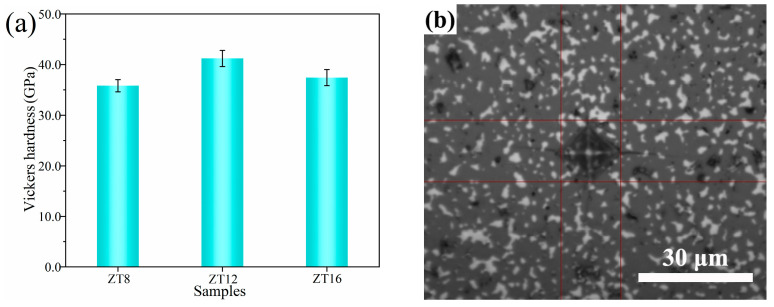
Vickers hardness and schematic image of the indentation of composite ceramics: (**a**) Vickers hardness, (**b**) schematic image.

Figure 9 shows the Vickers hardness and the indentation morphology of B_4_C–(Zr, Ti)B_2_ composite ceramics. It can be seen from Figure 9a that the Vickers hardness of the sample increases at first and then decreases, when n(B_4_C):n(Zr):n(Ti) = 12:1:1, the Vickers hardness reaches 41.2 GPa. The clear diamond-shaped indentations and extension cracks can be observed in Figure 9b. Combined with the BSE images (Figure 7), when the amount of Zr/Ti is reduced, the generation of (Zr, Ti)B_2_ is diminished, weakening the pinning effect on the B_4_C matrix—resulting in the coarsening of B_4_C grains, the reduction of the grain boundaries, and the reduction of the ability to resist local deformation. Ti^2+^ solid solution into the ZrB_2_ lattice will generate compression stress to enhance the internal energy. The active (Zr, Ti)B_2_ grains formation will block the movement of the crystal world in the sintered process of the SPS, reducing the grain size, which plays the role of good crystal reinforcement, because internal stress will be generated around the grains when the solid solution is formed. Compared with the mechanical properties data of B_4_C composite ceramics reported in references (Listed in Table 1), such as higher temperature and longer holding time, this work realizes the preparation of high hardness B_4_C ceramics at low temperatures. The Vickers hardness is better than the data reported in the references due to the solution strengthening effect of ZrB_2_ and TiB_2_.

**Figure 10:**
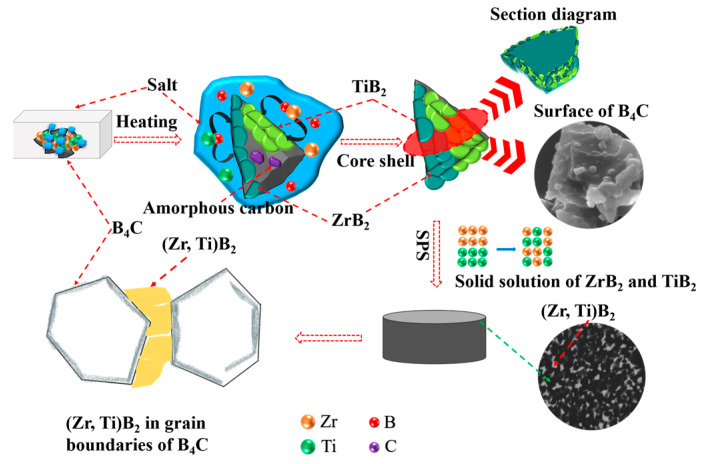
The mechanism diagram of the composite powder and ceramics.

Through the XRD phase analysis and microstructure of the composite powder and composite ceramics, and the ternary phase diagram of the B_4_C–Zr–Ti system at 1700 °C, the reaction mechanism of composite powder and ceramic was been studied: The mass transfer of Zr and Ti could be promoted because of the existence of a molten salt medium, Zr and Ti would react with B_4_C to form ZrB_2_ and TiB_2_ at high temperature and the product would coat the B_4_C surface. Thus, the composite powder exhibited a core–shell structure. When the composite powder was sintered by high temperature and pulse activation into composite ceramics, ZrB_2_ and TiB_2_ of the powder surface would produce a solid solution (Zr, Ti)B_2_). These (Zr, Ti)B_2_ solid solutions would prevent the movement of crystal boundaries during SPS sintering, thereby reducing the grain size of the composite ceramics. The reaction mechanism is shown in Figure 10.

## 4. Conclusions

B_4_C–(Zr, Ti)B_2_ composite ceramics were obtained by SPS sintering of the composite powder synthesized by the molten salt method, and its microstructure and mechanical properties were studied. The following conclusions were drawn.

(1) Thermodynamic calculation and phase analysis revealed that Zr and Ti reacted with B_4_C to form ZrB_2_ and TiB_2_ in a B_4_C-rich environment. Thanks to the heat and mass transfer of molten salt medium, the product is uniformly coated on the surface of B_4_C particles.
(2) When the powder is sintered by SPS at 1700 ℃, ZrB_2_ and TiB_2_ form solid solution (Zr, Ti)B_2_ at high temperature, the primary phases of composite ceramics are B_4_C, (Zr, Ti)B_2_, and C.
(3) Due to the synergistic effect of solid solution strengthening and particle toughening, better comprehensive properties can be obtained. When n(B_4_C):n(Zr):n(Ti) = 12:1:1, the Vickers hardness reach 41.2 GPa.

## Figures and Tables

**Table 1:** Mechanical properties data of B_4_C ceramics reported in references.

Sintering Process	Vickers Hardness (GPa)	References
Pressureless–sintered, 2100 °C, 60 min	30.2	[36]
Pressureless–sintered, 2100 °C, 60 min	34.2	[37]
Pressureless–sintered, 2150 °C, 60 min	33.2	[12]
HP, 1950 °C, 30 MPa, 60 min	34.6	[38]
HP, 1950 °C, 30 MPa, 60 min	–	[39]
HP, 1950 °C, 30 MPa, 30 min	35.22	[9]
HP, 2000 °C, 30 MPa, 60 min	33	[4]
SPS, 1700 °C, 32 MPa, 10 min	31.28	[5]
SPS, 2000 °C, 30 MPa, 3 min	32.33	[40]
SPS, 1700 °C, 50 MPa, 6 min	41.2	This work

## Data Availability

Data available on request due to restrictions privacy. The data presented in this study are available on request from the corresponding author.
